# Supplementary material for: OsLPR5 Encoding Ferroxidase Positively Regulates the Tolerance to Salt Stress in Rice
Source: Int J Mol Sci. 2023 Apr 30;24(9):8115. doi: 10.3390/ijms24098115 (PMC10179522; doi:10.3390/ijms24098115)
Supplement: Supplementary file 1 [file ijms-24-08115-s001.zip › ijms-2319292-supplementary.pdf]

Supporting Information

OsLPR5 encoding ferroxidase positively regulates the tolerance to salt stress in rice

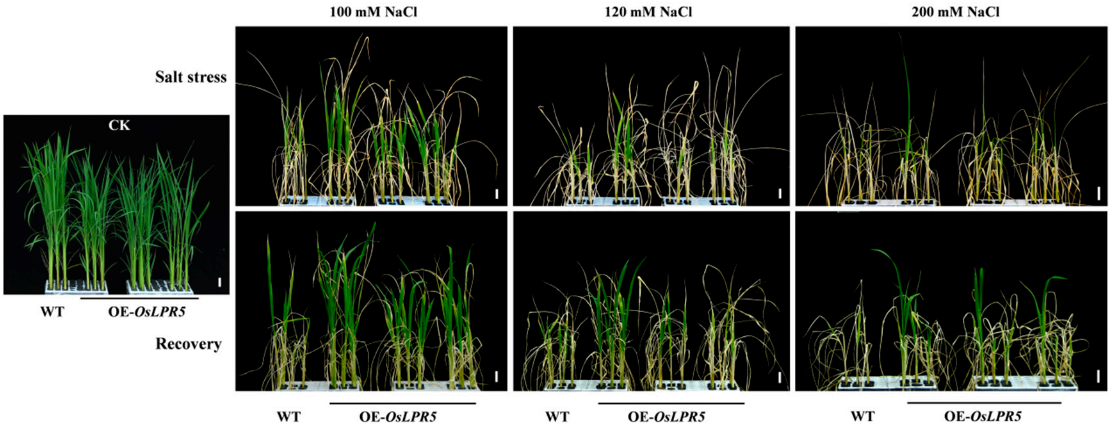

Figure S1. Overexpression of *OsLPR5* enhances the salinity tolerance of rice at the seedling stage exposed to 100, 120 and 200 mM NaCl stress for 7 days (Scale bars, 2 cm).

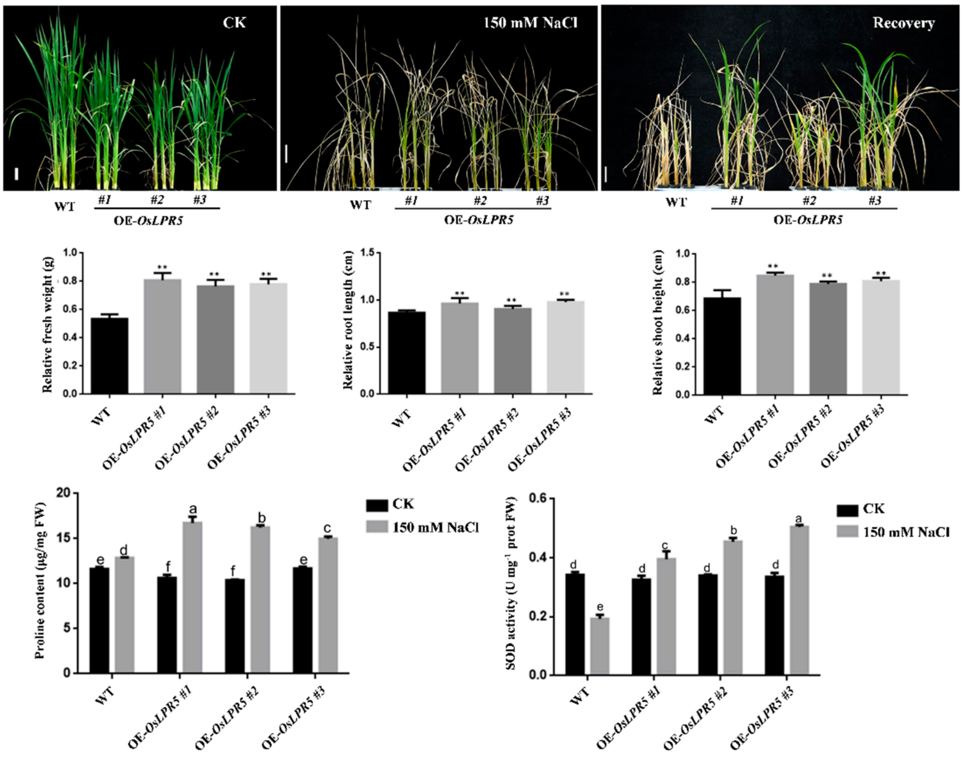

Figure S2. Overexpression of *OsLPR5* enhances the salinity tolerance of rice at the seedling stage exposed to 150 mM NaCl stress for 7 days (Scale bars, 2 cm). Different letters indicate significant differences between means as determined using ANOVA followed by Duncan’s test (P<0.05).

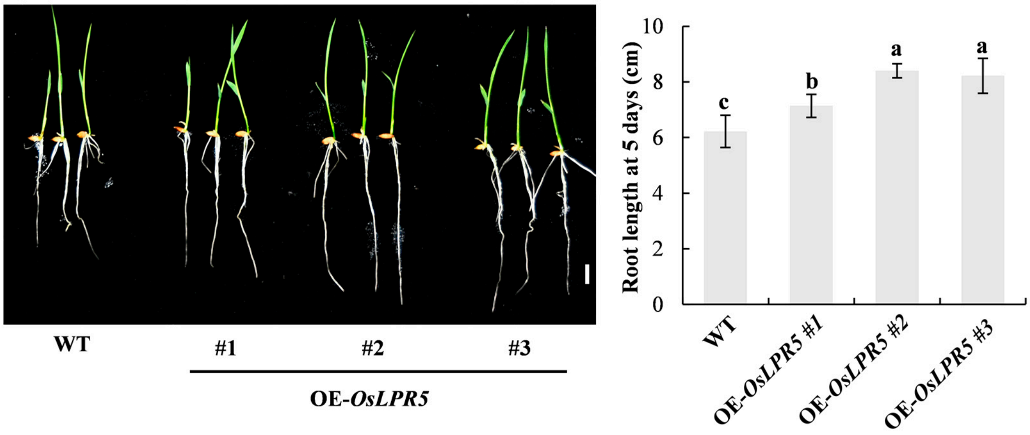

Figure S3. Overexpression of *OsLPR5* promotes the length of the primary after germination and growth for 5 days compared with wild type (WT) (Scale bars, 2 cm). Different letters indicate significant differences between means as determined using ANOVA followed by Duncan's test (*P*<0.05).

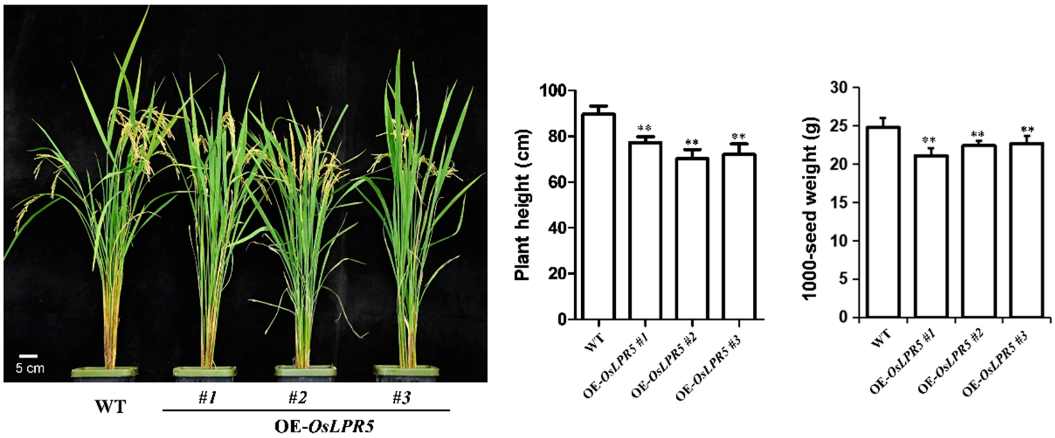

Figure S4. The photograph, plant height and 1000-grain weight of wild type and OsLPR5 overexpressed plants in the mature stage. Asterisk represents statistical difference at ***P* < 0.01 by t-test.

Table S1. Primers used for vector construction and qRT-PCR.

**Primer**	**Sequence (5'-3')**
OsLPR5-RT-F	CGAGGGATTCCAGGTGTTCAAG
OsLPR5-RT-R	GATCCAGGATGTGGCAGTGGTAG
OsLPR5-U3-F	ggcaACAGCCGCCAACAACACAG

OsLPR5-U3-R	aaacCTGTGTTGTTGGCGGCTGT
OsLPR5-OE-F	agctcggtacccggggatcctctagaATGAGTCCGAGGATTCAGC
OsLPR5-OE-R	tcctcgcccttgctcaccatgtcgacTGGAAGCAGCTTCAGAGGC
OsP5CS1-RT-F	TCTGCTCAGTGATGTGGATG
OsP5CS1-RT-R	CCTACACGAGATTTGTCTCC
OsYSL15-RT-F	GCGTTCGCCGTGCTCACGAACGTGG
OsYSL15-RT-R	ATCCTCCACCCATGAAATTAAACAC
OsHKT2;1-RT-F	CGGCGTTTCTGGCATCA
OsHKT2;1-RT-R	GCACCGAACAATGTGACCAA
